# Endocrine Dysfunction in Primary Mitochondrial Diseases

**DOI:** 10.1210/endrev/bnaf002

**Published:** 2025-02-01

**Authors:** Rachel Varughese, Shamima Rahman

**Affiliations:** Department of Endocrinology, Great Ormond Street Hospital for Children NHS Foundation Trust, London WC1N 3JH, UK; Mitochondrial Research Group, Genetics and Genomic Medicine Department, UCL Great Ormond Street Institute of Child Health, London WC1N 1EH, UK; Metabolic Department, Great Ormond Street Hospital for Children NHS Foundation Trust, London WC1N 3JH, UK

**Keywords:** primary mitochondrial disease, maternally inherited diabetes and deafness, Kearns-Sayre syndrome, adrenal insufficiency, premature ovarian insufficiency, Perrault syndrome

## Abstract

Primary mitochondrial disorders (PMD) are genetic disorders affecting the structure or function of the mitochondrion. Mitochondrial functions are diverse, including energy production, ion homeostasis, reactive oxygen species regulation, antioxidant defense, and biosynthetic responsibilities, notably including steroidogenesis. Mitochondria provide the energy to drive intracellular production and extracellular secretion of all hormones. The understanding of the endocrine consequences of PMD is key to timely identification of both endocrine complications in PMD patients, and PMD presenting primarily with endocrine disease. This is a narrative review on the endocrine manifestations of PMD, underlying disease mechanisms, and current and emerging approaches to diagnosing and treating these complex disorders. Diabetes is the most frequent endocrine manifestation of PMD, but growth hormone deficiency, adrenal insufficiency, hypogonadism, and parathyroid dysfunction may occur. Despite the intricate involvement of the thyroid gland in metabolic regulation, there is little evidence for a causal relationship between thyroid dysfunction and PMD. In conclusion, endocrine dysfunction is observed in PMD with varying incidence depending on the specific mitochondrial disorder and the endocrine organ in question. Diagnosis of PMD in a patient with endocrine-presenting features requires a high level of clinical suspicion, particularly when apparently unrelated comorbidities co-exist. Similarly, endocrine pathology may be subtle in patients with known PMD, and thorough consideration must be given to ensure timely diagnosis and treatment. The scope for novel therapeutics for this group of devastating conditions is enormous; however, several challenges remain to be overcome before hopes of curative treatments can be brought into clinical practice.

## Graphical Abstract

**Graphical Abstract:**
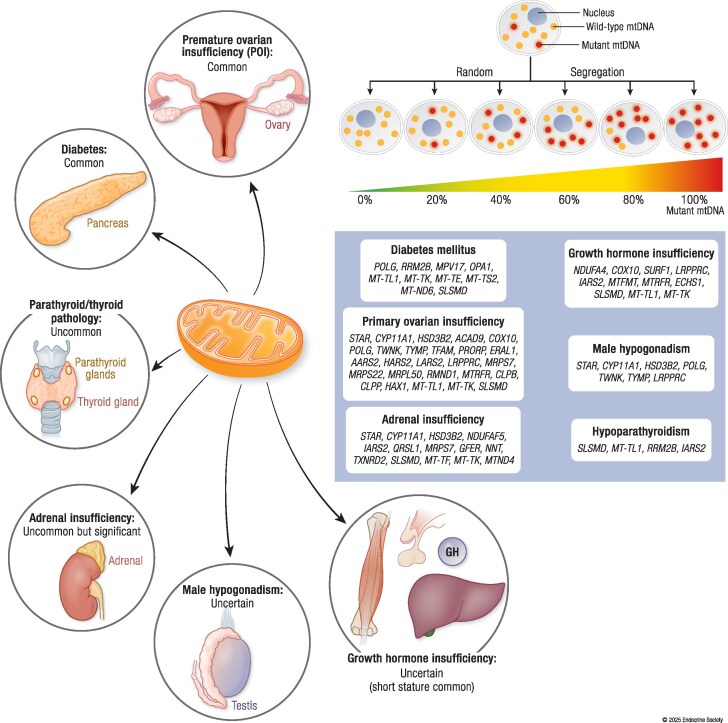

**Essential points**

- Primary mitochondrial disorders are genetic disorders that can arise from diverse patho-mechanisms, including disrupted oxidative phosphorylation and defects of mitochondrial DNA maintenance, protein synthesis, membrane lipids, protein quality control, organelle dynamics, and antioxidant defense
- Mitochondria house steroidogenesis and provide the energy to drive intracellular production and extracellular secretion of all hormones
- Diabetes mellitus is the most frequent endocrine manifestation of mitochondrial disease, but growth hormone deficiency, adrenal insufficiency, hypogonadism and parathyroid dysfunction also occur
- Endocrine dysfunction may be the primary presentation of mitochondrial disease, before neurological features arise, but more commonly occurs in the context of complex multisystem disease
- A high index of clinical suspicion of an underlying mitochondrial disorder is needed in a patient presenting with multisystemic disease and endocrine abnormalities

Mitochondrial diseases are a diverse group of disorders that arise from dysfunction in the mitochondria, the energy-producing powerhouses within our cells. Mitochondria are present in all cells except mature erythrocytes, and they provide a multitude of functions (1). The result of mitochondrial dysfunction is a widely heterogenous set of disorders. This means virtually any organ in the body can be affected, leading to a complex clinical, biochemical, and genetic picture. The understanding of primary mitochondrial disease has been greatly improved by next-generation sequencing, with more than 400 genes across 2 genomes now implicated.

Endocrine dysfunction is well understood to be linked to mitochondrial dysfunction, as mitochondrial functions often underpin hormone production, hormone secretion, and homeostatic sensing mechanisms. Theoretically any endocrine organ can be affected by mitochondrial disease, although certain tissues seem to be more susceptible.

## Review of Normal Mitochondrial Function

**Figure 1.**
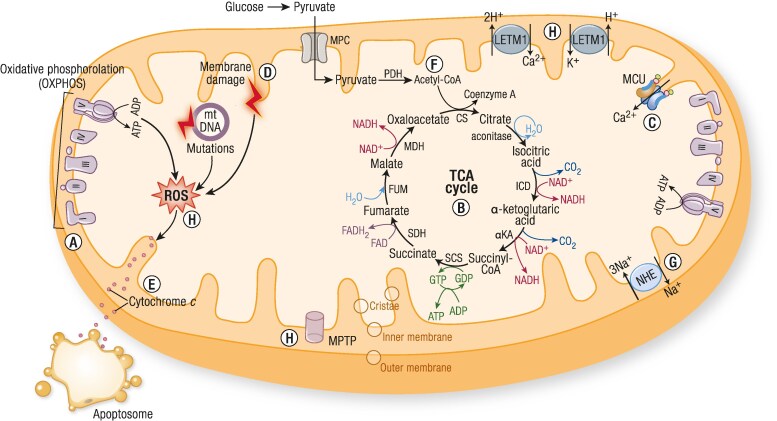
The multiple functions of mitochondria. A, *Energy production:* A primary function of mitochondria is to generate adenosine triphosphate (ATP), the energy currency of the cell, through a process called oxidative phosphorylation. This occurs in the inner mitochondrial membrane, where electrons derived from the breakdown of nutrients (such as glucose and fatty acids) are transferred through the respiratory or electron transport chain comprising 4 enzyme complexes (Complexes I-IV), coupled to the synthesis of ATP by Complex V (the ATP synthase). B, *Cellular respiration:* In addition to oxidative phosphorylation, mitochondria also participate in other metabolic pathways, such as the Krebs tricarboxylic (citric) acid cycle and fatty acid beta-oxidation, to extract energy from various fuel sources. C, *Calcium homeostasis:* Mitochondria play a critical role in maintaining cellular calcium homeostasis. They sequester and release calcium ions, which are involved in cellular signaling and regulate various cellular processes, including muscle contraction, neurotransmitter release, and cell death. Complex mechanisms govern the influx and efflux of calcium including the NCLX (sodium/lithium/calcium exchanger), LETM1 (leucine-zipper and EF-hand-containing transmembrane protein 1) and MCU (mitochondrial calcium uniporter), as well as the mitochondrial permeability transition pore, a calcium-dependent, ion nonselective membrane pore. D, *Reactive oxygen species (ROS) regulation:* Mitochondria are a major source of ROS, which are natural by-products of cellular respiration. While ROS can have harmful effects on cells, mitochondria have defense mechanisms, such as antioxidants and enzymes, to regulate ROS levels and maintain cellular redox balance. E, *Apoptosis regulation:* Mitochondria are involved in regulating programmed cell death, known as apoptosis. They release signaling molecules, such as cytochrome *c*, which activate the apoptotic pathway and play a crucial role in removing damaged or unwanted cells during development, tissue homeostasis, and disease processes. F, *Lipid metabolism:* Mitochondria are involved in various lipid metabolic pathways, including fatty acid beta-oxidation, ketone body synthesis, and cholesterol metabolism. They contribute to the synthesis, breakdown, and transport of lipids within cells, and to the biosynthesis of steroid hormones. G, *Metabolite and ion transport:* Mitochondria facilitate the exchange of metabolites, ions and cofactors between the cytoplasm and mitochondrial matrix, enabling the movement of molecules required for energy production and other metabolic processes. H, *Cell signaling:* Mitochondria participate in cellular signaling pathways by releasing signaling molecules, such as ROS and calcium ions, which can modulate cellular responses, gene expression, and cell fate decisions. Abbreviations: cytC, cytochrome c; LETM1, Leucine-zipper and EF-hand-containing TransMembrane protein 1; MCU, mitochondrial calcium uniporter; MPC, Mitochondrial pyruvate carrier; mPTP, mitochondrial permeability transition pore); NCLX, sodium/lithium/calcium exchanger; PDH, pyruvate dehydrogenase; ROS, reactive oxygen species; TCA, tricarboxylic acid.

Mitochondria are dynamic double-membraned organelles found in most human cells (all except mature erythrocytes), which lie at the center of cell metabolism and intracellular and extracellular signaling. They perform a myriad of essential functions (Fig. 1), which explains the diversity of disease when these processes are disrupted.

## Definition of Primary Mitochondrial Disease

Primary mitochondrial disorders (PMDs) are defined as genetic disorders affecting the structure or function of the mitochondrion (2), with an estimated birth prevalence of at least 1 in 4300 (3). Mitochondrial functions include production of energy by oxidative phosphorylation (OXPHOS), calcium homeostasis, generation of reactive oxygen species (ROS), regulation of apoptosis, antioxidant defense, anaplerosis, and diverse biosynthetic functions, including synthesis of membrane lipids, iron-sulfur clusters and heme, and, importantly, steroidogenesis (4, 5). Mitochondria not only house steroid hormone biosynthesis but are also essential for the supply of energy to drive the intracellular production and extracellular secretion of all hormones.

## Genetics and Inheritance of Primary Mitochondrial Disease

PMDs comprise monogenic disorders of 2 genomes: the nuclear genome and the dedicated small circular mitochondrial genome (mitochondrial DNA, mtDNA) located within the mitochondria themselves. The mitochondrial genome is unique in that it is exclusively maternally inherited (6, 7). The mtDNA exists as a circular DNA molecule that encodes 13 OXPHOS proteins as well as 2 ribosomal RNA (rRNA) and 22 transfer RNA (tRNA) molecules essential for intramitochondrial synthesis of these 13 proteins.

**Figure 2.**
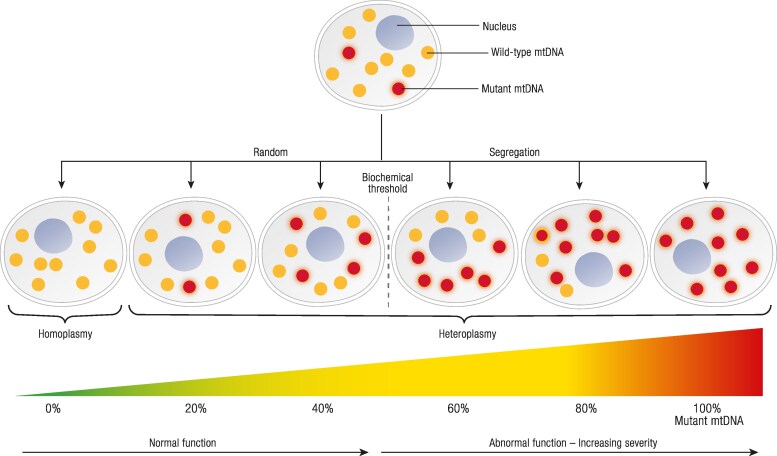
*Heteroplasmy* refers to the co-existence of wild-type and mutant mitochondrial DNA (mtDNA) molecules within cells, which varies among offspring due to variable segregation during mitosis. A small portion of mutant mtDNA may not result in mitochondrial dysfunction. The biochemical threshold describes the percentage of mutant mtDNA which will result in abnormal function. Cells with higher energy requirements (eg, brain and muscle) may have a lower biochemical threshold.

The genetics of mtDNA disease are complicated by the concepts of homoplasmy and heteroplasmy (Fig. 2). Since cells contain a high copy number of mtDNA (up to hundreds or thousands of copies depending on the cell type), cells may be homoplasmic (where 100% of mtDNA copies are the same genotype), or heteroplasmic (where mutant and wild-type mtDNA co-exist). This means mtDNA mutations can exist in different proportions within a person's cells, resulting in intragenerational and intergenerational variability. The severity of mtDNA-related PMD is influenced by a concept known as the threshold effect. Cells require a minimum threshold of mutated mtDNA to manifest clinically (8). If the mutated mtDNA levels exceed this threshold, cellular function is compromised, leading to disease symptoms. Interestingly, the specific threshold level required to cause symptoms varies depending on the specific pathogenic mtDNA variant and the affected tissue or organ. This leads to the potential for co-existence of severely affected and completely unaffected organs (9).

Approximately 7% of the nuclear exome encodes proteins targeted to mitochondria (10) including the majority of the subunits of the OXPHOS enzymes and dozens of proteins required for their coordinated assembly, together with proteins needed for mtDNA maintenance and gene expression (which together number into the hundreds—including numerous transcription and translation factors and ∼80 mitochondrial ribosomal proteins), mitochondrial import proteins, and enzymes involved in fatty acid oxidation, the Krebs cycle, and biosynthesis of lipids including steroid hormones, coenzyme Q_10_, heme, iron-sulfur clusters, and other cofactors. Unlike mtDNA mutations, pathogenic variants in these nuclear genes follow classical Mendelian inheritance.

To date more than 400 genes have been identified to have pathogenic variants causing PMD. The dual genomic inheritance of mitochondria means that any mode of inheritance may apply: autosomal recessive, autosomal dominant, X-linked, maternal (for mtDNA defects), and sporadic. Pathological mechanisms leading to PMD include defects of subunits and assembly factors of the 5 OXPHOS enzyme complexes, impaired mtDNA maintenance and gene expression, defects of mitochondrial protein import, membrane lipid biosynthesis, cristae organization and organellar dynamics, and disorders of mitochondrial antioxidant defense.

## Epidemiological Considerations

A 2020 retrospective study analyzing clinical, biochemical, and genetic features of participants with PMDs enrolled in the North American Mitochondrial Disease Consortium (NAMDC) registry reported endocrine manifestations in 20% (81/404) of the cohort (11, 12). However, this may be an underestimate owing to incomplete ascertainment. Another possibility is that this is an overestimate of the overall prevalence since it is not possible to determine from the publication whether the same patients had multiple endocrine complications of their PMD. The NAMDC registry data also revealed a lower frequency of endocrine disorders in patients with pathogenic variants in nuclear DNA compared to those with mtDNA variants (9.1% vs 24.6%, *P* < .0001). This was particularly true for diabetes mellitus (2.8% vs 19.1%, *P* = .0001) (11).

## Why Mitochondrial Disease May Lead to Endocrine Dysfunction

**Figure 3.**
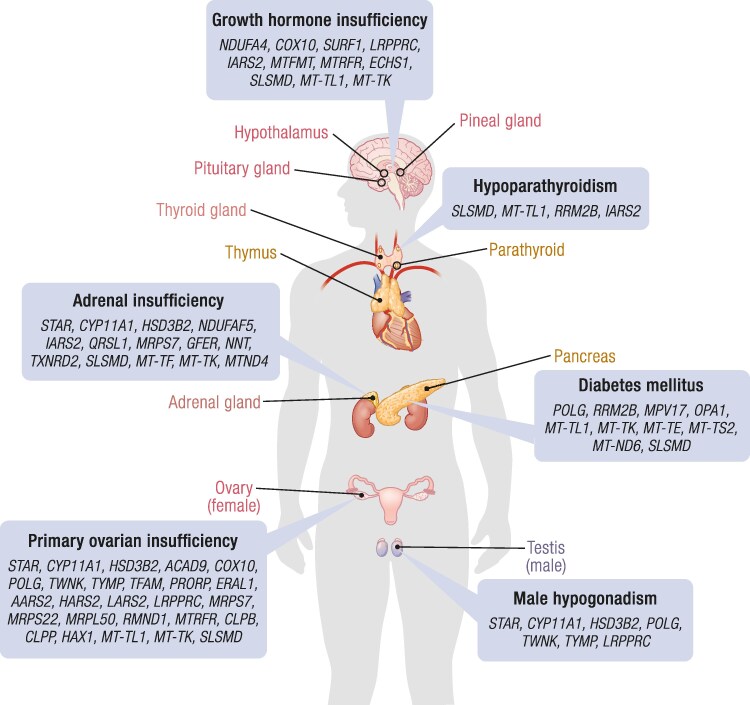
Endocrine organ involvement in primary mitochondrial disease and associated gene defects. Cartoon illustrating gene defects associated with dysfunction of endocrine organs in individuals with primary mitochondrial disease. Abbreviation: SLSMD, single large-scale mitochondrial DNA deletion.

Endocrine dysfunction is well understood to be commonly linked to mitochondrial dysfunction, as mitochondrial functions underpin most hormone production, secretion, and homeostatic sensing mechanisms. Dysfunction of any endocrine organ may occur in PMD (Fig. 3). Although endocrine dysfunction most commonly occurs in the context of a multisystem presentation, it can be the first clinical feature of PMD. Awareness of this possibility among endocrinologists should help to expedite diagnosis of mitochondrial disease and pave the way for the development of innovative therapies.

Mitochondria play essential roles in steroid biosynthesis. All steroid hormones, including cortisol, aldosterone, estrogen, progesterone, and testosterone are synthesized within mitochondria; therefore, lack of ATP because of mitochondrial dysfunction can lead to impaired hormone production. Cholesterol serves as the precursor for steroid hormone synthesis. Mitochondria receive cholesterol from the cytoplasm via protein transporters, such as the steroidogenic acute regulatory protein (StAR). StAR facilitates the transport of cholesterol from the outer mitochondrial membrane to the inner mitochondrial membrane. Within mitochondria, cholesterol is converted into pregnenolone, the initial step in steroidogenesis. This conversion involves the enzymatic activity of cholesterol side-chain cleavage enzyme (CYP11A1), which is localized in the inner mitochondrial membrane. This enzymatic reaction requires electron transfer from the respiratory chain within the mitochondria. Pregnenolone can be converted into different steroid hormones depending on the specific cell type and the enzymatic machinery present. For example, in the adrenal cortex, cortisol and aldosterone are synthesized through a series of enzymatic reactions within the mitochondria. Similarly, in the gonads, mitochondria are involved in the synthesis of estrogen, progesterone, and testosterone.

## Mitochondrial Disease Mechanisms

### Disorders of OXPHOS Complex Subunits and Assembly Factors

Disorders of OXPHOS complexes and their assembly refer to a group of mitochondrial diseases characterized by defects in the 4 respiratory chain complexes (Complexes I-IV) and the ATP synthase (Complex V). These disorders can result from pathogenic variants in the 13 mtDNA genes encoding OXPHOS subunits or in more than a hundred nuclear genes encoding subunits or assembly factors of the OXPHOS complexes (13, 14).

### Disorders of Mitochondrial DNA Maintenance

Disorders of mtDNA maintenance refer to a group of nuclear gene–related PMDs characterized by defects in the replication, repair, or maintenance of mtDNA (15). These disorders can result in the progressive depletion of mtDNA or in the accumulation of mtDNA point variants or large-scale deletions. Responsible genes include *POLG* and *POLG2* encoding the catalytic and accessory subunits of polymerase gamma (16), the only DNA polymerase that can replicate the mtDNA, and *TWNK* encoding the Twinkle helicase-primase needed to unwind mtDNA prior to replication. Defects in the mitochondrial nucleoside salvage apparatus may also impair mtDNA replication, because of a lack of building blocks for mtDNA synthesis (17).

### Disorders of Mitochondrial Protein Synthesis

Mitochondrial protein synthesis disorders can broadly be categorized into 2 main groups: (i) mtDNA-related disorders; and (ii) nuclear DNA-related disorders (18, 19). The first includes disorders involving the mitochondrial tRNAs such as mitochondrial encephalomyopathy, lactic acidosis, and stroke-like episodes (MELAS) syndrome, and myoclonic epilepsy with ragged red fibers (MERRF), or disorders associated with single large-scale mtDNA deletions (SLSMDs) such as Pearson marrow pancreas and Kearns-Sayre syndromes and progressive external ophthalmoplegia (PEO). The latter includes defects of genes encoding tRNA modification factors, aminoacyl tRNA synthetases (enzymes that attach specific amino acids to cognate tRNAs to allow protein synthesis), mitochondrial ribosomal proteins, and factors required for initiation, elongation, and termination of mitochondrial translation. Impaired mitochondrial protein synthesis leads to a deficiency of essential components of the OXPHOS system, with consequent effects on cellular energy production.

The clinical presentation of SLSMD disorders, including Pearson and Kearns-Sayre syndromes, can exhibit extreme variability, both in terms of the affected tissues or organs and the severity of symptoms. This variability is partly due to the heteroplasmy phenomenon, where cells can have a mixture of normal and mutated mtDNA (20). The degree of heteroplasmy, or the proportion of mutant mtDNA within a cell, can greatly influence the severity of symptoms. This is not always the case—for example, most cases of adrenal insufficiency in SLSMD syndromes have been shown to have no correlation between deletion size and presentation severity/age. In addition, varying energy demands across different tissues means that the impact of mitochondrial dysfunction can be more pronounced in tissues with high energy demands, such as the brain, muscles, and heart. Some SLSMD disorders exhibit a threshold effect, where symptoms become evident only when the level of mutated mtDNA surpasses a certain threshold in specific tissues (21). Additionally, the interplay between genetic factors, including the specific location and size of mtDNA deletions, and environmental factors can contribute to clinical variability. Some SLSMD disorders may present with age-related variability in symptoms. Certain tissues may be more vulnerable to mitochondrial dysfunction as individuals age, leading to the emergence of new symptoms or the worsening of existing ones over time (22).

### Disorders of the Mitochondrial Membranes

Mitochondrial membranes are essential for many cellular processes, including as physical anchors for the OXPHOS enzyme complexes and supercomplexes, energy production, protein import, regulation of metabolite influx into and efflux from the mitochondria, and crosstalk with other cellular organelles through membrane contact sites (23).

#### Lipid disorders

Mitochondrial membranes contain several different phospholipids, including some that are common to other cell membranes, namely phosphatidylcholine, phosphatidylethanolamine, phosphatidylinositol, phosphatidylserine, phosphatidic acid, and phosphatidylglycerol (24). A highly specialized lipid found exclusively in the inner mitochondrial membrane is cardiolipin, whose unique structure confers physicochemical properties essential for mitochondrial function (25). Disruptions in the synthesis or metabolism of these lipids can impact membrane structure and function and cause PMD.

#### Cristae organization disorders

Cristae are invaginations of the inner mitochondrial membrane that house the machinery for OXPHOS; therefore, disorders affecting cristae structure may impact mitochondrial respiration and ATP production. A relatively recent advance in the mitochondrial field has been the discovery of the mitochondrial contact site and cristae organizing system (MICOS) complex (26-30), and defects of MICOS proteins are emerging as causes of multisystem PMDs.

#### Protein import disorders

More than 1000 mitochondrial proteins are encoded by nuclear DNA and synthesized on cytosolic ribosomes. Thus, effective protein import into mitochondria is essential for maintaining mitochondrial function (31). Mutations in genes encoding components of the translocase of the inner mitochondrial membrane (TIM) and translocase of the outer mitochondrial membrane (TOM) complexes can impair protein import, known collectively as TIM and TOM complex deficiencies (32). Protein import involves recognition of proteins destined for the mitochondria, which contain specific targeting signals recognizable to the TOM complex. Once recognized, proteins pass through the outer mitochondrial membrane via the TOM complex and then through the inner mitochondrial membrane via the TIM complex. Proteins are further processed and sorted within the mitochondria to reach their final destinations, either within the mitochondrial membranes, in the intermembrane space, or in the mitochondrial matrix. TIM and TOM complex deficiencies may result in a range of PMDs affecting multiple organ systems.

### Disorders of Mitochondrial Protein Quality Control

Mitochondrial protein quality control is a critical cellular process that involves monitoring, maintaining, and regulating the integrity of mitochondrial proteins (33, 34). Dysregulation or dysfunction of these quality control systems can lead to various disorders impacting mitochondrial function. There are several aspects of mitochondrial protein quality control that can be affected (35). This group is increasingly recognized to lead to mitochondrial dysfunction with endocrine involvement, for example deficiencies of the mitochondrial protease CLPP and the disaggregase CLPB have both been linked to premature ovarian insufficiency (POI).

#### Ubiquitin-proteasome system

The ubiquitin-proteasome system (UPS) is involved in the targeted degradation of misfolded or damaged proteins whereby proteins tagged with ubiquitin are recognized and degraded by the proteasome. Dysfunction of the UPS can result in the accumulation of misfolded or damaged mitochondrial proteins (36).

#### Mitochondrial proteases

Mitochondria contain specific proteases responsible for cleaving and degrading misfolded or unfolded proteins within the organelle (37, 38). Protease deficiencies or mutations can impair the removal of damaged proteins, leading to mitochondrial protein aggregation and dysfunction.

#### Chaperone-mediated protein folding

Molecular chaperones assist in the proper folding of mitochondrial proteins and prevent their aggregation (39). Mutations in chaperone proteins or deficiencies in their activity can result in accumulation of misfolded proteins that leads to impaired mitochondrial function.

#### Mitophagy

Mitophagy is the process by which damaged or dysfunctional mitochondria are selectively removed through autophagy. Impaired mitophagy can lead to the accumulation of damaged mitochondria, contributing to cellular dysfunction (40).

#### Mitochondrial unfolded protein response

Mitochondrial unfolded protein response (UPRmt) is a cellular response activated in response to mitochondrial protein misfolding or dysfunction. Dysregulation of UPRmt can impact the cell's ability to cope with mitochondrial protein stress (41).

### Disorders of Mitochondrial Dynamics

Disorders of mitochondrial dynamics involve abnormalities in the processes of mitochondrial fission, fusion, and motility. These dynamic processes are essential for maintaining a healthy mitochondrial network within cells.

#### Mitochondrial fission

Dynamin-Related Protein 1 (DRP1) is a protein involved in mitochondrial fission, the process of dividing mitochondria into smaller units (42). Excessive fission or impaired fusion can lead to fragmented mitochondria (43). Mutations or dysregulation of proteins involved in mitochondrial fission, such as DRP1 and the mitochondrial fission factor (MFF), can result in the accumulation of elongated or interconnected mitochondria.

#### Mitochondrial fusion

This process involves fusion of the mitochondrial membranes, allowing mitochondria to merge and share contents (43). Examples of fusion dysfunction occur in deficiency of MFN1/2 (Mitofusin 1 and 2), proteins that mediate the fusion of the outer mitochondrial membrane, and deficiency of OPA1 (Optic Atrophy 1), a protein required for inner mitochondrial membrane fusion. Pathogenic variants in *MFN2* or *OPA1* can lead to fragmented mitochondria and impaired fusion, affecting mitochondrial function and distribution (44, 45).

#### Mitochondrial motility

Mitochondrial motility requires intact microtubules to move along, and functioning motor proteins, such as kinesin and dynein, to facilitate this process. Impaired mitochondrial transport along microtubules can lead to uneven distribution and dysfunction. In addition, deficiencies in proteins responsible for anchoring mitochondria in specific cellular regions can affect their motility.

### Disorders of Mitochondrial Antioxidant Defense

Disorders of mitochondrial antioxidant defense refer to conditions where the normal balance between the production of ROS and the antioxidant defense mechanisms within the mitochondria is disrupted (46). Mitochondria are a major source of ROS, which are natural byproducts of cellular respiration (47). ROS, including superoxide and hydrogen peroxide, are normally produced at low levels. Mitochondria possess antioxidant systems, including enzymes such as superoxide dismutase, catalase, and glutathione peroxidase, to neutralize ROS and maintain redox balance. Mutations affecting genes encoding mitochondrial antioxidant enzymes or proteins involved in ROS regulation can lead to impaired antioxidant defense. Mutations in mtDNA can affect the respiratory chain, leading to increased ROS production and oxidative stress (48-51). Excessive ROS production or deficient antioxidant defense can result in oxidative stress, causing damage to proteins, lipids, and DNA within the mitochondria. Persistent oxidative stress can exacerbate mitochondrial dysfunction, compromising cellular energy production, in turn leading to further ROS production and a vicious spiral that contributes to the phenotypic manifestations of PMDs.

## Systems-Based Review of Endocrine Manifestations in PMD According to Endocrine Organ Affected

### Diabetes

Diabetes is the most frequently described endocrine manifestation of mitochondrial disease. This may be type 1 or type 2 diabetes mellitus (52), although in reality, it is mostly a mixed picture, presenting in a state of non-insulin dependence, with likely rapid progression to requirement for insulin replacement. This is sometimes referred to as *diabetes 1.5* in mitochondrial literature. Mitochondrial diabetes is particularly associated with the maternally inherited diabetes and deafness (MIDD) syndrome, mostly caused by a point variant in mtDNA m.3243A>G in *MT-TL1*. These individuals have a mixed picture of insulin deficiency and insulin resistance (53, 54). Prevalence of mitochondrial diabetes caused by this variant is reported from 0.5% (55) up to 2.9% in Japan (56), with an average age of diabetes onset of 38 years, although an older age of onset has been reported for some other mtDNA point mutations (57). The diabetes in MIDD is typically an adult phenomenon. In the pediatric population, SLSMDs presenting as Kearns-Sayre and Pearson syndromes are more common causes of mitochondrial diabetes, frequently as part of multisystem endocrine disease such as adrenal insufficiency, hypothyroidism, growth hormone deficiency, and hypoparathyroidism (58, 59).

#### Pathophysiology

The development of diabetes in mitochondrial disease is multifactorial. The frequently mixed type 1/type 2 picture points to depleted pancreatic beta cell function coupled with insulin resistance, for which risk factors such as hyperglycemia, central adiposity, and limited exercise are often present (60). Mitochondrial function, in particular OXPHOS, is vital in homeostatic mechanisms of glucose-stimulated insulin secretion. Mouse models demonstrate diminished insulin secretion in mice with decreased OXPHOS subunit expression (61). Both mtDNA and nuclear DNA variants, leading to decreased mitochondrial OXPHOS subunit expression and oxidative function as well as other disease mechanisms discussed above, can lead to the development of diabetes in human PMD (Table 1). Currently there are no available mouse models of m.3243A>G or SLSMDs, the most frequent causes of mitochondrial diabetes, to study the patho-mechanisms of mitochondrial diabetes related to these mtDNA variants directly. Mitochondrial transcription factor A (TFAM) is essential for mtDNA biogenesis and maintenance, with transcription factors B1 (TFB1M) and B2 (TFB2M) required for mtDNA transcription and translation. Knockout mice for any of these 3 genes develop diabetes mellitus, with impaired glucose-stimulated insulin secretion initially, followed by increasing beta cell loss with age (62-64). Interestingly, insulin secretory responses to arginine have been demonstrated to be normal in human patients with MIDD, presumably due to direct depolarization of the beta cell plasma membrane at neutral pH in the presence of glucose, bypassing mitochondrial OXPHOS (64, 65).

**Table 1.** Mitochondrial diabetes and associated gene defects

Implicated genes (variants)	Mode of inheritance	Other clinical features	Disease mechanism
***Nuclear genes***			
*POLG*	AD AR	PEO, ptosis, myopathy, ataxia, peripheral neuropathy, dysphagia	Impaired mtDNA maintenance
*RRM2B*	AD AR	PEO, ptosis, SNHL, proximal myopathy, ataxia, mood disturbance, GI dysmotility, sensory axonal peripheral neuropathy	Impaired mtDNA maintenance
*MPV17*	AR	Axonal sensorimotor peripheral neuropathy, ptosis, PEO, exercise intolerance, steatohepatopathy, depression, parkinsonism, GI dysmotility	Impaired mtDNA maintenance
*OPA1*	AD	Optic atrophy, PEO, SNHL, ataxia, myopathy, peripheral neuropathy	Impaired mtDNA maintenance
***Mitochondrial genes***			
*MT-TL1:* *m.3243A>G* *m.3254C>G* ^*a*^ *m.3256C>T* ^*a*^ *m.3264T>G* ^*a*^ *m.3271T>C*	Maternal	MIDD, MELAS, PEO, myopathy	Impaired tRNA function
*MT-TK (m.8344A>G, m.8356T>C)*	Maternal	MERRF	Impaired tRNA function
*MT-TE (m.14709T>C)*	Maternal	Myopathy, ataxia, encephalomyopathy	Impaired tRNA function
*MT-TS2: m.12258T>C* ^*a*^	Maternal	MIDD, cataracts, ataxia	Impaired tRNA function
*MT-ND6: m.14577T>C* ^*a*^	Maternal	Diabetes mellitus only	Impaired complex I
*SLSMD*	Sporadic	Pearson syndrome, Kearns-Sayre syndrome	Impaired translation

Abbreviations: AD, autosomal dominant; AR, autosomal recessive; GI, gastrointestinal; MELAS, mitochondrial encephalomyopathy with lactic acidosis and stroke-like episodes; MERRF, myoclonic epilepsy ragged red fibers; MIDD, maternally inherited diabetes and deafness; PEO, progressive external ophthalmoplegia; SLSMD, single large-scale mitochondrial DNA deletion; SNHL, sensorineural hearing loss.

^a^mtDNA variants reported but not confirmed pathogenic.

#### Maternally inherited diabetes and deafness

MIDD is a rare mitochondrial disorder characterized by a combination of diabetes mellitus and sensorineural deafness, both of which are passed down exclusively through the maternal lineage. Maternally inherited diabetes was first reported in association with a large mtDNA deletion in 1992, with a pedigree analysis by the Wallace laboratory providing landmark evidence of the importance of mtDNA heteroplasmy (66). The m.3243A>G variant is the more usual cause of MIDD and is one of the most well-known and prevalent mtDNA variants associated with MIDD, although other mtDNA variants can also be responsible. This specific variant occurs in the *MT-TL1* gene, which encodes a tRNA for leucine that recognizes the UUR codon. This mutation affects mitochondrial protein synthesis and thus disrupts OXPHOS and ATP production as a result of reduced OXPHOS subunit expression.

The hearing impairment in MIDD is symmetric and sensorineural, secondary to cochlear dysfunction, starting in young adulthood. Beyond diabetes and deafness, some individuals with MIDD may exhibit additional clinical features such as myopathy, cardiomyopathy, or neurologic abnormalities, underscoring the systemic nature of mitochondrial dysfunction.

Diabetes onset can occur anywhere from the second decade but a mean age of onset toward the end of the fourth decade was observed in several studies (57, 67, 68).

The m.3243A>G variant is highly prevalent in the population, present in 1 in 400 people (69), and associated phenotypes are highly variable, even within the same pedigree. Many individuals with this variant are asymptomatic, while those at the more severe end of the disease spectrum develop MELAS syndrome. In those with MIDD, it is difficult to predict who will develop insulin vs non-insulin dependent diabetes. MIDD patients are invariably nonobese, a finding conserved across multiple ethnicities, including Japanese (70), French (67), and UK populations (57). There is very limited evidence to suggest any autoimmune mechanism in MIDD, although occasionally autoantibodies may be detected, likely in patients with co-existing autoimmune diabetes. Two reviews examining populations of North European descent have reported that 13% of patients with m.3243A>G MIDD required insulin from diagnosis, with diabetic ketoacidosis reported at presentation in several cases (57). In both reviews, approximately half of the remaining patients progressed to insulin therapy, with half classified as non-insulin dependent. The heterogeneity persists across ethnicities into Asian and Black populations, although both of these tend to have a higher proportion with type 2 non-insulin dependent diabetes. The variability of MIDD is typified by the families exhibiting both insulin dependent (presenting in diabetic ketoacidosis) and non-insulin dependent diabetes across first-degree relatives (68, 71, 72).

The hearing loss in MIDD often precedes the diagnosis of diabetes (57) and so a history of bilateral sensorineural hearing loss (SNHL) in a patient with new onset diabetes would be a clear indication to investigate for MIDD.

#### Diabetes associated with SLSMDs

Pearson marrow pancreas syndrome is a rare mitochondrial disorder that affects multiple systems in the body, particularly the bone marrow and exocrine pancreas. It is an early-onset SLSMD disorder, often presenting with transfusion-dependent sideroblastic anemia and lactic acidosis, named after the British pediatric hematologist and geneticist Richard Pearson, who first described the syndrome in the 1970s (73), although it was not until 9 years later that it was attributed to mtDNA deletion (74). Along with clinical features including bone marrow dysfunction, growth failure, feeding difficulties, lactic acidosis, neurological dysfunction (seizures, global developmental delay), and liver abnormalities, exocrine pancreatic insufficiency is a frequent feature. Endocrine insufficiency is also described in some patients, whereby insulin dependent diabetes mellitus may exist independently of exocrine abnormalities, with onset as early as the neonatal period. The pathogenesis is not clear, but postmortem examinations have revealed pancreatic atrophy and fibrosis in 2 patients from 2 separate reports, with hypoplasia in another (59, 75, 76). There is a high early childhood mortality rate in Pearson syndrome. Those who survive typically experience resolution of transfusion-dependent anemia, reflecting clearance of SLSMDs from rapidly dividing marrow cells, but go on to develop Kearns-Sayre syndrome. Kearns-Sayre syndrome, another SLSMD disorder, can also present in childhood or early adult life without preceding features of Pearson syndrome. Kearns-Sayre syndrome is defined clinically by a triad of PEO with onset prior to 20 years and one or more of: pigmentary retinopathy, cerebellar ataxia, elevated cerebrospinal fluid protein (>100 mg/dL), and/or heart block. Diabetes mellitus is a frequent complication of Kearns-Sayre syndrome, first reported in 1978 (77). The prevalence of mitochondrial diabetes in SLSMDs was reported to be 26% in a multicenter pediatric study and 9% in an adult cohort (59, 78).

#### Gestational diabetes

The association between mitochondrial disease and gestational diabetes mellitus (GDM) is unclear. One study from 2000 demonstrated several mtDNA variants (m.3398T>C, novel heteroplasmic m.3254C>A and homoplasmic m.3399A>T, and m.3316G>A and m.3394T>C mutations implicated in non-insulin dependent diabetes mellitus) to be seen in higher frequencies in patients with GDM than in control subjects, suggestive of mtDNA contribution to GDM in some patients (79). However, these findings have not been validated in the genomic era, and these may merely represent benign population variants in the mtDNA. Another study postulated that body mass index modulates mitochondria due to increased oxidative stress, leading to mitochondrial dysfunction, linking this to GDM onset (80). More obviously, and unsurprisingly, there is evidence of an association between MIDD and GDM. A recent Irish cohort study retrospectively studied the obstetric and perinatal outcomes in 88 pregnancies of 26 women with genetically confirmed mitochondrial disease, 25 of whom had m.3243A>G (81). GDM was observed in 21/88 (24%) of these pregnancies. This is higher than in previous retrospective cohort studies, such as reported by Janssen et al, who in 2015 examined 98 pregnancies across 46 women with m.3243A>G, finding GDM prevalence of 11% (82). Risk of GDM is particularly relevant, as metformin treatment should be approached with caution in patients with MIDD due to the risk of lactic acidosis. There was an association of MIDD and miscarriage above the general population average. Modulating the obstetric risk of women with PMD requires more knowledge about the specific obstetric complications. A limiting factor will always be that some diagnoses are made later in life, after affected women have already had children.

### Growth Hormone Deficiency

Short stature is a common manifestation of PMD, frequently reported in those with cytochrome *c* oxidase (complex IV) deficiency, MELAS, MERRF, and Kearn-Sayres syndromes, among other mitochondrial defects (83-86). As expected, the earlier in childhood the onset of a mitochondrial disease, the greater the likelihood of developing short stature, as demonstrated in a Japanese population comparing those with juvenile-onset MELAS (60%) to adult-onset (29%) (87). Variation in incidence of growth hormone deficiency in other PMDs cannot simply be explained by age of disease onset and requires further study. For example, short stature was far more prevalent (95%) in patients with Leigh syndrome (subacute necrotizing encephalomyelopathy) with complex IV assembly defect caused by surfeit locus protein 1 (SURF1) deficiency (88), compared to 52% in complex IV deficiency caused by variants in *LRPPRC* which encodes a protein that plays a role in mitochondrial RNA metabolism (85). Table 2 lists mitochondrial and nuclear gene defects reported to cause mitochondrial growth hormone insufficiency.

**Table 2.** Mitochondrial growth hormone insufficiency or short stature and associated gene defects

Implicated genes (variant)	Mode of inheritance	Other clinical features	Disease mechanism	Documented growth hormone deficiency
***Nuclear genes***				
*NDUFA4*	AR	Leigh syndrome	Impaired complex IV	Not documented
*COX10*	AR	Leigh syndrome, proximal renal tubulopathy, anemia, SNHL, nystagmus, hypertrophic cardiomyopathy	Impaired complex IV assembly	Not documented
*SURF1*	AR	Leigh syndrome	Impaired complex IV assembly	Not documented
*LRPPRC*	AR	Leigh syndrome (French Canadian)	Impaired translation	Not documented
*IARS2*	AR	Cataracts, sensory neuropathy, SNHL, skeletal dysplasia	Impaired translation	GH deficiency documented in several cases (89)
*MTFMT*	AR	Leigh syndrome, optic atrophy, WPW	Impaired translation	GH deficiency documented in 1 case (90)
*MTRFR*	AR	Leigh syndrome, optic atrophy, ophthalmoplegia, spastic paraplegia	Impaired translation	Not definitively documented; 2 patients treated with GH for 2 years but no response (91)
*ECHS1*	AR	Leigh syndrome	Toxic damage to thiols	Not documented
***Mitochondrial genes***				
*SLSMD*	Sporadic	Pearson syndrome, Kearns-Sayre syndrome	Impaired translation	GH deficiency documented in several cases (92-97)
*MT-TL1 (m.3243A>G)*	maternal	MELAS	Impaired tRNA function	GH deficiency documented (98-100)
*MT-TK (m.8344A>G)*	maternal	MERRF	Impaired tRNA function	GH deficiency documented in an unpublished case in the authors’ cohort

Abbreviations: AD, autosomal dominant; AR, autosomal recessive; GH, growth hormone; MELAS, mitochondrial encephalomyopathy with lactic acidosis and stroke-like episodes; MERRF, myoclonic epilepsy ragged red fibers; SLSMD, single large-scale mitochondrial DNA deletion; SNHL, sensorineural hearing loss; WPW, Wolff-Parkinson-White syndrome.

#### Pathophysiology

The pathophysiology of short stature in PMD is multifactorial. Growth hormone deficiency is described in multiple case reports of PMD, including MELAS (9, 98, 99) and SLSMDs (92, 101). Hypothalamic-pituitary axis dysfunction is supported by case reports of patients with multiple anterior pituitary hormone deficiencies (9, 102-104). One report demonstrated high levels of mutated mtDNA in the hypophysis of a patient with MELAS and growth hormone deficiency, suggesting mitochondrial energy deficiency as the cause, but definitive evidence of the mechanistic cause is lacking (9). Conversely, many patients have normal growth hormone stimulation studies, and brain imaging frequently shows normal pituitary appearances.

#### Other causes of mitochondrial short stature

Aside from growth hormone deficiency, several other mechanisms could account for short stature in PMD. Infants with mitochondrial dysfunction are often born smaller than healthy comparators due to compromised prenatal growth, seen most strikingly in those with maternal m.3243A>G disease (105-108). As with many chronic diseases, growth can be adversely influenced in PMD owing to disease-related inadequate protein substrate. Nutrition plays a major role in this and on its own can account for low insulin-like growth factor 1 levels. Gastrointestinal pathology such as gastroparesis, dysphagia, reflux, and intestinal pseudo-obstruction, all frequent comorbidities of PMD, can reduce dietary intake. Moreover, the defective energy production from a given nutritional unit that underpins mitochondrial disease may exacerbate inadequate nutritional delivery. Co-existing issues, such as renal impairment, are also important factors, as are neuromuscular manifestations leading to impaired muscle mass, skeletal load, and longitudinal bone growth.

### Adrenal Insufficiency

Steroidogenesis relies on mitochondria for generation of cellular ATP and their ability to house steroidogenic reactions. However, only a fraction of PMDs features adrenal insufficiency, predominantly occurring in SLSMDs such as Pearson and Kearns-Sayre syndromes, although within these disorders, adrenal dysfunction is still rare.

#### Pathophysiology

Cortisol is a glucocorticoid hormone produced in the zona fasciculata layer of the adrenal cortex, essentially implicated in stress, inflammation, and immune responses, as well as glucose and protein homeostasis. Cortisol dysfunction can be lethal, and prompt identification is vital to ensure that adequate replacement is provided when needed. As previously discussed, critical elements of steroidogenesis occur within mitochondria.

Despite this, mitochondrial diseases featuring adrenal insufficiency are rare, with fewer than 30 genetically confirmed cases reported in the literature (109). Adrenal dysfunction was documented in only 3 of 404 individuals in the NAMDC mitochondrial cohort (12), although this may be an underestimate, reflecting incomplete ascertainment and reporting and/or inconsistent testing of adrenal function in patients known to have a PMD. The pathogenesis correlating these genetic changes with the mechanism of cortisol deficiency remains challenging to elucidate. It is unclear why some patients develop adrenal insufficiency while others do not; even hypotheses implicating the burden of mutated mitochondria (heteroplasmy level) in the specific tissues is heavily debated, with adrenal insufficiency prevailing in the face of relatively low mtDNA mutational burden compared to other organs that appear clinically unaffected (93).

#### Familial glucocorticoid deficiency

Approaching 50% of familial glucocorticoid deficiency (FGD) is caused by pathogenic variants in *MC2R* encoding the melanocortin receptor or in *MRAP* coding for the MC2R accessory protein (110). Isolated cortisol deficiency has also been reported as the clinical presentation associated with deficiencies of 4 mitochondrial proteins: StAR, CYP11A1, nicotinamide nucleotide transhydrogenase (NNT), and thioredoxin reductase 2 (TXNRD2) (109). Mutations in *STAR*, encoding a protein that shuttles cholesterol across the mitochondrial membranes, typically cause lipoid congenital adrenal hyperplasia with disordered sexual development, though milder defects may present as FGD. As discussed above, CYP11A1 catalyzes the conversion of cholesterol to pregnenolone, the first step of steroidogenesis. Mutations in *CYP11A1* result in similar clinical features to STAR deficiency, and together these disorders account for ∼10% of FGD. NNT is required to replete the mitochondrial NADPH pool to maintain redox balance and TXNRD2 is also involved in antioxidant defense. NNT and TXNRD2 deficiencies represent ∼10% of FGD (109). NNT and TXNRD2 deficiencies have so far only been reported to cause isolated cortisol deficiency and have not been linked to other manifestations of PMDs, but it is possible that more severe mutations in these genes may lead to a multisystem clinical presentation. Deficiency of another enzyme involved in redox homeostasis, ferrodoxin reductase (encoded by *FDXR*), causes a neurological PMD but has so far not been associated with cortisol deficiency.

#### Other causes of mitochondrial adrenal dysfunction

Of the reported cases, most affected individuals had SLSMDs presenting as Pearson or Kearns-Sayre syndromes, with variable mtDNA deletion sizes ranging from 1.5 to 9 kb. In addition, 2 patients had atypical MELAS syndrome: one with the m.8344A>G variant in *MT-TK* more usually associated with myoclonus epilepsy with ragged red fibers (MERRF) (111), and one patient with an *MT-ND4* variant (m.12015T>C;p.Leu419Pro) with atypical MELAS combined with comorbid polyglandular autoimmune syndrome type 2 including autoimmune adrenal disease. Adrenal dysfunction has also been reported in several nuclear-encoded PMDs, including a complex I assembly defect and disorders of mitochondrial translation and protein import (Table 3). Disease onset was hugely variable, ranging from 7 months to 32 years.

**Table 3.** Mitochondrial adrenal insufficiency and associated gene defects

Implicated genes	Mode of inheritance	Other clinical features	Disease mechanism
***Nuclear genes***			
*STAR*	AR	Lipoid congenital adrenal hyperplasia, gonadal dysgenesis, FGD	Impaired mitochondrial steroidogenesis
*CYP11A1*	AR	Lipoid congenital adrenal hyperplasia, FGD	Impaired mitochondrial steroidogenesis
*HSD3B2*	AR	Congenital adrenal hyperplasia	Impaired mitochondrial steroidogenesis
*NDUFAF5*	AR	IUGR, dysmorphism, lethal neonatal disease^*a*^	Impaired complex I assembly
*IARS2*	AR	Cataracts, sensory neuropathy, SNHL, skeletal dysplasia, GH insufficiency	Impaired translation
*QRSL1*	AR	Lethal neonatal disease, poor growth, hypertrophic cardiomyopathy, SNHL	Impaired translation
*MRPS7*	AR	SNHL, lactic acidosis, primary hypogonadism^*a*^	Impaired translation
*GFER*	AR	Lactic acidosis, congenital cataracts, respiratory insufficiency^*a*^	Impaired mitochondrial import
*NNT*	AR	FGD	Metabolism of ROS
*TXNRD2*	AR	FGD	Metabolism of ROS
***Mitochondrial genes***			
*SLSMD*	Sporadic	Pearson syndrome, Kearns-Sayre syndrome	Impaired translation
*MT-TF*	Sporadic	Developmental regression, lactic acidosis, hypotonia, GI dysmotility, end-stage renal disease^*a*^	Impaired tRNA function
*MT-TK (m.8344A>G)*	Maternal	MELAS/MERRF^*a*^	Impaired tRNA function
*MT-ND4 (m.12015T>C)*	Maternal	Atypical MELAS^*a*^	Impaired complex I

Abbreviations: AD, autosomal dominant; AR, autosomal recessive; FGD, familial glucocorticoid deficiency; GH, growth hormone; GI, gastrointestinal; IUGR, intrauterine growth restriction; MELAS, mitochondrial encephalomyopathy with lactic acidosis and stroke-like episodes; MERRF, myoclonic epilepsy ragged red fibers; ROS, reactive oxygen species; SLSMD, single large-scale mitochondrial DNA deletion; SNHL, sensorineural hearing loss.

^a^Single case reports.

#### Diagnostic suspicion

The rarity of adrenal insufficiency in people with SLSMDs or point variants in mtDNA or nuclear genes associated with PMD poses a diagnostic challenge. Adrenal symptoms can be nonspecific and blend into other systemic complaints, and under-detection and under-reporting may play a factor. There is no universally accepted guideline for screening for adrenal dysfunction in PMD populations; this may be reasonable given the likely low pick-up rate, but this may well lead to under-detection. Exome and genome sequencing is not always undertaken in patients presenting with adrenal dysfunction, although this is changing as these resources become more widely available, meaning that pathogenic changes in nuclear genes known to cause primary adrenal failure may remain unidentified. Importantly, adrenal insufficiency may be the presenting feature of PMD, with hyponatremia, hyperpigmentation, or adrenal crisis. A high index of suspicion of an underlying mitochondrial disease is needed.

### Hypogonadism

Hypogonadism is a known manifestation of mitochondrial disorders, affecting males and females, either as hypergonadotropic hypogonadism (ie, gonadal insufficiency) or hypogonadotropic hypogonadism (ie, neuroendocrine insufficiency).

In females, POI is very well characterized in women with PMDs; for example, in Perrault syndrome and other mitochondrial syndromes such as ovarioleukodystrophy caused by pathogenic variants in *AARS2* (see below).

The understanding of male hypogonadism in PMDs is much less clear than for females. Men with Perrault syndrome caused by PMD gene variants, or with *AARS2* variants, have not been reported to have oligospermia or azoospermia. However, it is important to note that since POI and SNHL are the 2 cardinal features of Perrault syndrome, there is likely to be an ascertainment bias where men are not diagnosed, mislabeled as having nonsyndromic SNHL, creating a void of knowledge when it comes to understanding their puberty and fertility status. Men with mitochondrial neurogastrointestinal encephalomyopathy (MNGIE), a disorder of mtDNA maintenance caused by thymidine phosphorylase deficiency, can present with both hypogonadotropic and hypergonadotropic hypogonadism (112, 113). Hypergonadotropic hypogonadism has also been reported in males with coenzyme Q_10_ deficiency (although the genetic cause remained elusive) (114) and the French Canadian variant of Leigh syndrome (85). Further clarification is required to confirm an association between *POLG* variants and male hypogonadism. Despite several reports of hypergonadotropic hypogonadism associated with *POLG* variants (115, 116) including a description of testicular atrophy (117) and an assertion that *POLG* contributes to at least 5% to 10% of cases of male infertility, spermatogenesis has conversely been demonstrated as normal across several studies (118-120). Other studies have been unable to confirm effects of *POLG* variants on male fertility, as they were all female cohorts (16).

#### Pathophysiology

The hypogonadism can broadly be divided into POI (defined by the loss of normal ovarian function before the age of 40), testicular dysfunction, and neuroendocrine dysfunction affecting the hypothalamic-pituitary axis. Gonadal dysfunction may occur in mitochondrial steroidogenic defects such as CYP11A1 and StAR deficiencies, due to impaired adrenal and gonadal steroidogenesis, as discussed above. Hypogonadotropic hypogonadism is particularly associated with SLSMDs such as Kearns-Sayre syndrome. One study demonstrated that around a fifth of patients with SLSMDs had evidence of hypogonadotropic hypogonadism, equally split between males and females (86). Hypogonadism may be complete, but is more often partial, with some spontaneous puberty and development of secondary sexual characteristics, followed by pubertal arrest or secondary amenorrhea associated with low gonadotropin levels (121-123). Hypergonadotropic hypogonadism has been associated with mtDNA maintenance disorders, such as pathogenic variants in the *TWNK* gene that encodes the mitochondrial Twinkle helicase, needed to unwind mtDNA prior to replication (124).

#### Fertility

Small cohorts of patients with certain pathogenic variants make analysis of fertility according to mechanistic subtype extremely difficult. A UK cohort of females with mitochondrial disease did not show any difference in fertility rate compared to general population averages, even in those severely affected, a surprising finding given that POI is a clearly documented feature of several PMDs, perhaps owing to the possibility of symptom onset after child-bearing age (125). It is even more difficult to attribute male infertility to mitochondrial dysfunction.

#### POI in mitochondrial disease

POI has been known under many names over the years, previously referred to as *primary ovarian insufficiency*, *premature/primary ovarian failure*, and less commonly *premature/early menopause*, *gonadal dysgenesis*, *hypogonadotropic hypogonadism*, *ovarian dysgenesis*, *hypergonadotropic amenorrhea*, *climacterium praecox*, or *menopause praecox*. The European Society of Human Reproduction and Embryology (ESHRE) guidelines in 2015 defined POI as a clinical syndrome comprising loss of ovarian activity before the age of 40, characterized by menstrual disturbance (amenorrhea or oligomenorrhea) with raised gonadotropins and low estradiol.

The etiology of POI is heterogenous, with acquired causes including autoimmune disease, infection, and medication side effects. Up to 30% of patients with POI have at least one affected relative (126, 127). Twin and family history studies exhibit clear heritability, highlighting the presence of genetic factors, some 90 of which have been identified since the prevalent implementation of next-generation sequencing (128). A 2005 study examining menopausal age within the multigenerational female participants of the Framingham Heart study reported that at least 50% of predisposition for menopausal age could be accounted for by genetic heritability (129). However, the majority of women with POI remain without a genetic diagnosis, highlighting the scope for future genetic discovery that remains.

Recently, PMDs were reported in 11.9% of solved cases (23/193) in a large cohort of 1030 Chinese women with POI. PMD genes reported to cause POI in the Chinese cohort were *POLG*, *TWNK*, *AARS2*, *HARS2*, *MRPS22*, *CLPP*, *ACAD9*, and *COX10* (130). The involvement of mitochondrial dysfunction in POI is intuitive. Compared with all cell types across the body, human oocytes contain the largest number of mitochondria (131, 132). Functional mitochondrial biosynthesis underpins successful oocyte maturation and fertilization. The relationship of POI with PMDs is also associated with the influence of ROS in regulation of sex hormone synthesis and follicular growth in ovarian tissue, with evidence that women with POI exhibit elevated oxidative stress markers (133-135). Impaired follicular oocyte development follows this oxidative stress, leading to early depletion of the quantity and quality of the primordial follicular pool. Ovarian aging is further accelerated by follicular depletion by way of oocyte apoptosis, reducing ovarian function. Conversely, normal levels of ROS regulate healthy follicular growth, intrathecal angiogenesis, and sex hormone synthesis (136).

PMD genes associated with POI are responsible for a variety of functions, including involvement in metabolism, meiosis, DNA repair, mitochondrial function, and follicular development. These genes include *POLG*, *TWNK*, *ERAL1*, *AARS2*, *HARS2*, *LARS2*, *MRPS22*, *LRPPRC*, and *CLPP* (Table 4). *POLG* encodes the catalytic subunit of DNA polymerase gamma, the only polymerase able to replicate the mtDNA, and has been particularly linked to POI. *POLG* variants were reported to cause POI or primary amenorrhea, associated with neurological features including PEO, sensory ataxia, parkinsonism, and muscle weakness, in 8 women from 4 families (137). In 3 of these families, the genetic cause was a dominant p.Tyr955Cys *POLG* variant, affecting the catalytic polymerase domain (POL B motif) of the polymerase gamma enzyme.

**Table 4.** Mitochondrial ovarian insufficiency and associated gene defects

Implicated genes	Mode of inheritance	Other clinical features	Disease mechanism
***Nuclear genes***			
*STAR*	AR	Lipoid congenital adrenal hyperplasia	Impaired mitochondrial steroidogenesis
*CYP11A1*	AR	Lipoid congenital adrenal hyperplasia	Impaired mitochondrial steroidogenesis
*HSD3B2*	AR	Congenital adrenal hyperplasia	Impaired mitochondrial steroidogenesis
*ACAD9*	AR	Hypertrophic cardiomyopathy, lactic acidosis, exercise intolerance	Impaired complex I assembly
*COX10*	AR	Leigh syndrome, proximal renal tubulopathy, anemia, SNHL, nystagmus, hypertrophic cardiomyopathy	Impaired complex IV assembly
*POLG*	AD or AR	PEO, ataxia neuropathy spectrum, parkinsonism	Impaired mtDNA maintenance
*TWNK*	AR	Perrault syndrome, PEO, ataxia, peripheral neuropathy	Impaired mtDNA maintenance
*TYMP*	AR	MNGIE	Impaired mtDNA maintenance
*TFAM*	AR	SNHL, seizures, intellectual disability	Impaired mtDNA maintenance and transcription
*PRORP*	AR	Perrault syndrome, leukoencephalopathy, lactic acidosis	Impaired mitochondrial tRNA processing
*ERAL1*	AR	Perrault syndrome	RNA chaperone defect
*AARS2*	AR	Leukodystrophy, ataxia, spasticity, cognitive decline	Impaired translation
*HARS2*	AR	Perrault syndrome	Impaired translation
*LARS2*	AR	Perrault syndrome	Impaired translation
*LRPPRC*	AR	Leigh syndrome	Impaired translation
*MRPS7*	AR	SNHL, liver and renal failure	Impaired translation
*MRPS22*	AR	Cardiomyopathy, hypotonia, lactic acidosis, dysmorphism, Leigh syndrome spectrum	Impaired translation
*MRPL50*	AR	SNHL, renal impairment, LVH	Impaired translation
*RMND1*	AR	SNHL, chronic kidney disease	Impaired translation
*MTRFR*	AR	Leigh syndrome, optic atrophy, ophthalmoplegia, spastic paraparesis	Impaired translation
*CLPB*	AR	Cataracts, neutropenia, intellectual disability	Impaired mitochondrial quality control
*CLPP*	AR	Perrault syndrome, leukodystrophy	Impaired mitochondrial quality control
*HAX1*	AR	Severe congenital neutropenia	Impaired mitochondrial membrane potential
***Mitochondrial genes***			
*MT-TL1 (m.3243A>G)*	Maternal	MELAS, MIDD	Impaired tRNA function
*MT-TK*	Maternal	MERRF	Impaired tRNA function
*SLSMD*	Sporadic	Kearns-Sayre syndrome	Impaired translation

Abbreviations: AD, autosomal dominant; AR, autosomal recessive; LVH, left ventricular hypertrophy; MELAS, mitochondrial encephalomyopathy with lactic acidosis and stroke-like episodes; MERRF, myoclonic epilepsy ragged red fibers; MIDD, maternally inherited diabetes and deafness; MNGIE, mitochondrial neurogastrointestinal encephalomyopathy; PEO, progressive external ophthalmoplegia; SLSMD, single large-scale mtDNA deletion; SNHL, sensorineural hearing loss.

Based on these robust associations between *POLG* and POI, 2 studies sought to establish the prevalence of *POLG* disease in women with POI by screening large cohorts with POI for common pathogenic *POLG* variants, but only one *POLG* variant was found in 258 women across the 2 studies (138, 139). Therefore, while *POLG* is clearly associated with POI, the unlikelihood of isolated POI means that *POLG* disease should primarily be considered in women with other features of mitochondrial disease, particularly neurological complaints including PEO, parkinsonism, seizures, neuropathy, and ataxia (16).

#### Perrault syndrome

First described by French physicians Perrault and Kurz in 1951, Perrault syndrome is a rare autosomal recessive (usually) mitochondrial disorder characterized by a combination of 2 main features: POI in females (seen to varying degrees of severity, with onset in adolescence or early adulthood) and bilateral progressive SNHL. In addition to these primary features, individuals with Perrault syndrome may also exhibit other clinical manifestations, although these are less consistent, including muscle weakness, intellectual disability, cerebellar ataxia, sensory peripheral neuropathies, and leukodystrophy. Perrault syndrome is genetically heterogeneous with pathogenic variants reported in *TWNK* (encoding the mitochondrial Twinkle helicase needed to unwind mtDNA prior to replication), *ERAL1* (encoding a mitochondrial rRNA chaperone) (140), *HARS2* (encoding mitochondrial histidyl tRNA synthetase) (141), *LARS2* (encoding mitochondrial leucyl tRNA synthetase) (142), and *CLPP* (encoding an ATP-dependent mitochondrial protease required for UPRmt, an essential component of the mitochondrial quality control system) (143). The exact mechanisms linking these genetic mutations to the development of POI and SNHL in Perrault syndrome are not fully understood. Variants in *HSD17B4*, encoding a peroxisomal enzyme 17β-hydroxysteroid dehydrogenase type 4, involved in fatty acid β-oxidation and steroid metabolism, represent the only nonmitochondrial cause of Perrault syndrome identified to date. Despite recent genetic advances in this syndrome, the majority of patients with Perrault syndrome remain without a molecular diagnosis.

#### Other mitochondrial causes of syndromic POI

Several studies have reported patients with primary hypogonadism associated with SNHL and other clinical features extending beyond Perrault syndrome, including a homozygous variant in *MRPS7* encoding mitochondrial ribosomal protein S7 in siblings with congenital SNHL and lactic acidemia. One of these siblings died age 14 years from hepatic and renal failure without reported pubertal status, but the second sister developed POI with primary amenorrhea. MRPS7, a 12S ribosomal RNA-binding subunit of the small mitochondrial ribosomal subunit, is required for the assembly of the small ribosomal subunit. Another small mitochondrial ribosomal subunit, MRPS22, was previously linked to fatal infantile disease characterized by severe hypotonia, hypertrophic cardiomyopathy, and lactic acidosis. Subsequently, homozygous *MRPS22* variants were reported in 4 individuals from 2 consanguineous families who presented with isolated POI in adolescence (144). A Drosophila model highlighted the importance of MRPS22 in ovarian development (144). More recently, a 2023 study demonstrated a link between a homozygous missense variant in *MRPL50* shared by twin sisters, who both presented with POI and bilateral SNHL, with accompanying renal and cardiac dysfunction (145). The resulting MRPL50 protein loss led to destabilization of the large subunit of the mitochondrial ribosome. Both knockout and knockdown of MRPL50 in Drosophila confirmed abnormal ovarian development, as for MRPS22.

Ovarioleukodystrophy, which refers to POI associated with progressive leukodystrophy (white matter neurological abnormalities, particularly involving the myelin sheath), is a relatively recent term, first described in 1997 (146). This was initially linked to pathogenic variants in 3 eukaryotic translation initiation factor 2B (EIF2B) genes (147, 148), but in 2014 was first reported in a PMD, caused by pathogenic variants in *AARS2* encoding mitochondrial alanyl tRNA synthetase (149). Prior to this, *AARS2* variants had primarily been associated with fatal infantile cardiomyopathy (150), highlighting the phenotypic variability of PMDs.

POI has also been reported in 3 girls with French Canadian Leigh syndrome caused by deficiency of leucine-rich pentatricopeptide repeat domain containing protein (LRPPRC, which functions as a de-adenylase involved in mitochondrial mRNA transport and stability), all of whom had accompanying POI that responded well to estrogen treatment (151). Leigh syndrome has been linked to defects in at least 113 genes (152), but POI is not a frequently reported feature, perhaps because of the high mortality rate of this condition in early childhood (153).

### Parathyroid Dysfunction

Hypoparathyroidism is not a common finding across many PMDs (Table 5); however, it is consistently reported in Kearns-Sayre syndrome resulting from SLSMDs (8) and can even be the presenting feature (154, 155), with reports of tetanic hypocalcemia (86). Hypomagnesemia, a common accompanying and possible driving feature of hypoparathyroidism, is present in some children (156, 157) but not others (158, 159) and can be related to Gitelman-type renal tubular dysfunction with inappropriate magnesuria. It has also been postulated that hypomagnesemia may drive the hypoparathyroidism through parathyroid hormone suppression; however, magnesium supplementation does not reverse the biochemical picture. Moreover, serum phosphate is often noted to be elevated, rather than the low-normal picture seen in magnesium deficiency–induced hypoparathyroidism. The hypothesis that atrophy of parathyroid glands may underpin hypoparathyroidism is supported in autopsy reports of absent parathyroid glands (160) but is harder to explain in autopsies where even one histologically normal parathyroid gland remains (158). Case reports reflect an increased incidence of other endocrine abnormalities in Kearns-Sayre syndrome when hypoparathyroidism is present; however, the bias of historically more robust endocrine investigations in those with hypoparathyroidism is difficult to account for, as acknowledged in a 1992 case review and literature survey (86). Despite this, several reports link hypoparathyroidism in Kearns-Sayre syndrome to diabetes mellitus and other multisystem disease (161, 162), suggesting a propensity to be associated with a more severe clinical picture. The pathogenic reasons behind this are unclear, with a high parathyroid heteroplasmic threshold being a possible cause. Autoimmune etiology has not been proven.

**Table 5.** Mitochondrial hypoparathyroidism and associated gene defects

Implicated genes (variant)	Mode of inheritance	Other clinical features	Disease mechanism
***Mitochondrial genes***			
*SLSMD*	Sporadic	Kearns-Sayre syndrome	Impaired translation
*MT-TL1 (m.3243A > G)*	Maternal	MIDD	Impaired tRNA function
***Nuclear genes***			
*RRM2B*	AR	PEO, ptosis, SNHL, proximal myopathy, peripheral neuropathy, renal failure	Impaired mtDNA maintenance
*IARS2*	AR	Sideroblastic anemia, seizures, DD (single case report)	Impaired translation

Abbreviations: AR, autosomal recessive; DD, developmental delay; MIDD, maternally inherited diabetes and deafness; PEO, progressive external ophthalmoplegia; SLSMD, single large-scale mitochondrial DNA deletion.

### Thyroid Dysfunction

While there have been occasional case reports documenting hypothyroidism in patients with PMD, particularly in those with SLSMDs, for example in patients with Kearns-Sayre syndrome (92), there is insufficient evidence to support a causal association. A 2020 study concluded that the prevalence of hypothyroidism was no higher than the population average in a North American PMD cohort (11). Another obvious confounding factor is the possibility of the presence of undetected subclinical or mild clinical hypothyroidism, leading to under-reporting. Conversely, screening for thyroid dysfunction in patients with known molecular diagnoses of PMD could lead to artificially inflating the frequency of hypothyroidism, compared to an unscreened general population.

## Diagnostic Approach

**Figure 4.**
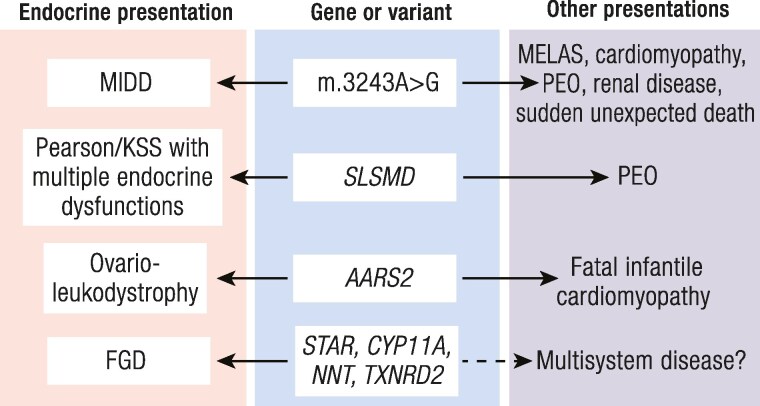
Phenotypic heterogeneity in primary mitochondrial disease. *Phenotypic heterogeneity* describes the variation in disease characteristics among individuals who share the same genetic mutation. Genes and variants shown in blue in middle of cartoon, with associated endocrine features to the left (in pink) and other clinical features associated with the same gene/variant to the right. The dotted arrow represents the possibility that genes currently associated with isolated familial glucocorticoid deficiency may theoretically cause multisystem disease, although this has not been demonstrated in patients to date.

The diagnostic approach to PMDs should encompass a consideration of clinical features, imaging findings, and the results of biochemical investigations. Strict pathognomonic features for mitochondrial disease do not exist owing to the wide range of clinical features and severity observed in PMDs, likely reflecting the heterogeneous genetic mechanisms and mitochondrial pathways impacted by these diseases. It is probably more likely that endocrine abnormalities will be identified in patients with known mitochondrial disease, than mitochondrial diseases identified in those first presenting with endocrine dysfunction. There is no accepted consensus on screening for endocrine pathology in mitochondrial disease, but some recommendations have been made by an international consortium (163). Since numbers are small for each endocrine problem and mitochondrial condition, the yield is likely to be low. Identifying mitochondrial disease in the endocrine clinic is more challenging, and a high level of clinical suspicion is therefore mandatory, particularly for more common conditions such as diabetes mellitus. Endocrinologists need to remain vigilant when there is an atypical presentation of diabetes mellitus, short stature, adrenal insufficiency, POI, or hypoparathyroidism/hypocalcemia, and interrogate for history of suspicious features such as neurological complaints (previous stroke-like episodes, seizures, ataxia, ophthalmoplegia, ptosis) or biochemical features of lactic acidosis, unexplained hypoglycemic episodes, or abnormal plasma amino acid, acylcarnitine, or urinary organic acid profiles. Although classical syndromes featuring particular constellations of symptoms and signs are recognized (eg, MIDD, MELAS, MERRF, Pearson and Kearns-Sayre syndromes) the majority of patients do not present with a typical clinical syndrome, especially in childhood. More subtle presentations may include the linking of 2 seemingly coincidental unrelated disorders, which may fall under one unifying mitochondrial umbrella. Frequently the possibility of a mitochondrial disorder is considered only when an endocrine problem is present with another more characteristic symptom of mitochondrial disease, such as PEO, or a symptom affecting a distant/unrelated organ system. Table 6 highlights some typical scenarios that should prompt investigation for an underlying PMD. A final problem, which is difficult to circumvent, is of the twin issues of phenotypic heterogeneity and phenocopies, that is, of nonmitochondrial diseases mimicking PMDs and associated with secondary mitochondrial dysfunction, leading to a wide differential diagnosis (Fig. 4).

**Table 6.** A clinical guide to diagnosis of mitochondrial endocrine disease

Endocrine feature	Additional feature(s)	Mitochondrial syndrome to consider	Associated PMD gene (variant)
Diabetes mellitus	Deafness (SNHL)	MIDD	*MT-TL1 (m.3243A > G)*
Diabetes mellitus	Sideroblastic anemia	Pearson	*SLSMD*
Adrenal insufficiency	Sideroblastic anemia	Pearson	*SLSMD*
Adrenal insufficiency	PEO, pigmentary retinopathy, heart block	Kearns-Sayre	*SLSMD*
Growth hormone deficiency	PEO, pigmentary retinopathy, heart block	Kearns-Sayre	*SLSMD*
Ovarian insufficiency	Deafness (SNHL)	Perrault	*TWNK, ERAL1*
Ovarian insufficiency	Leukodystrophy	Ovarioleukodystrophy	*AARS2, CLPP*
Ovarian insufficiency	Cataracts and 3MGA	CLPB deficiency	*CLPB*

Abbreviations: 3MGA, 3-methylglutaconic aciduria; MIDD, maternally inherited diabetes and deafness; PEO, progressive external ophthalmoplegia; PMD, primary mitochondrial disorder; SLSMD, single large-scale mitochondrial DNA deletion; SNHL, sensorineural hearing loss.

Muscle biopsy used to be the cornerstone of mitochondrial diagnosis but has been superseded by the widespread use of whole exome and whole genome sequencing in recent years, which provide a genetic diagnosis in 50% to 60% of cases with suspected mitochondrial disease. Testing of other tissues may be needed for mtDNA variants, which may be present at low heteroplasmy levels in blood. Negative genetic testing in serum samples in highly suspected mitochondrial disease requires consideration of follow up with urine mtDNA testing, with or without muscle testing due to the possible segregation of pathogenic variants across different tissues. Muscle biopsy is also useful for providing histological or biochemical evidence of mitochondrial dysfunction in cases with negative genetics.

## Approaches to Treatment

### Endocrine Management

The endocrine aspect of management is usually straightforward, requiring confirmation of hormone deficiency, followed by replacement and titration of doses to clinical effects and biochemistry. Patients with confirmed growth hormone deficiency have been treated with growth hormone with varying degrees of success, with several reports confirming absence of adverse effects (98, 164, 165). Growth hormone trials in those without confirmed growth hormone deficiency are not recommended due to potential adverse effects in this population. Side effects and mechanisms are only based on theory, with numbers too small for definitive conclusions. Potentially, the stimulation of cell proliferation and differentiation by growth hormone could expend essential energy required elsewhere, exacerbating an already energy-deficient state. There are infrequent reports of those whose condition worsened on treatment. One case report describes 2 cases of rapid clinical deterioration involving hypotonia and language deficit upon growth hormone commencement, which resolved on cessation of growth hormone therapy, postulated to be secondary to stimulation of mitosis increasing energy demand (166). Tentative conclusions regarding long-term outcomes of growth hormone therapy, even in those with confirmed growth hormone deficiency, are also suggestive of alternative underlying etiology for short stature, with some reports demonstrating initial effectiveness of growth hormone therapy, with absence of therapeutic benefit when observed over several years (167). There may be future potential for gonadotropin replacement in this patient population, with previous success using human chorionic gonadotropin treatment to restore testosterone levels and bring about secondary sexual characteristic maturation in a patient with Kearns-Sayre syndrome (168).

### Diabetes Mellitus

A comprehensive review of diabetes in mitochondrial disease has recently been published, with a suggested pathway for the management of patients with both insulin dependent and non-insulin dependent diabetes (60). A general rule is to manage these patients in the same way that they would be managed in the classical versions of these diseases. However, acknowledgment of the relatively high frequency of conversion from non-insulin dependent to insulin deficiency should prompt closer monitoring and safety netting for concerning symptoms and signs, together with testing for ketosis. As discussed above, metformin may not be the best treatment for mitochondrial diabetes, owing to the increased risk of lactic acidosis in this population. We therefore suggest that metformin therapy should only be considered for mitochondrial diabetes under specialist supervision where lactate levels are closely monitored, and with clear guidance as to when patients should seek advice for investigation or dose omission. Concerns regarding the exacerbation of neurological manifestations, particularly stroke-like episodes, following metformin treatment in patients with MIDD caused by the m.3243A>G variant have been reported (169). However, the small sample size and lack of objective data make it difficult to draw robust conclusions about the causal relationship between neurological decline and metformin treatment in this patient.

### Mitochondrial Disease Management

There is currently no cure for PMDs, and treatment options are predominantly focused on supportive management of symptoms and improving the quality of life for affected individuals (163). The natural history of disease is highly variable, with some genetic defects more predictably associated with certain serious sequelae than others. Anticipation of clinical manifestations facilitates appropriate safety netting and patient education of expected symptoms and signs, opening the door for early diagnosis and treatment.

### Experimental Therapies

The development of disease-modifying curative therapies for PMDs has been beset by several challenges, including the relative inaccessibility of the mitochondrion to drugs and genetic therapies, and a lack of suitable animal models to facilitate preclinical drug development and to evaluate the efficacy of genetic therapies (170). However, recent developments in base editing of mtDNA show promise in allowing the creation of mouse models for mtDNA disease (171). Other challenges are that it is unlikely that a single therapy will cure the more than 400 PMDs that are currently known, or even the multiple problems that an individual affected by a PMD may face.

Several novel experimental therapies are currently being explored in the field of mitochondrial diseases. Emerging experimental therapies can broadly be divided into pharmacological and genetic therapies and have been the subject of recent reviews (172, 173). Pharmacological approaches include antioxidants, mitochondrial membrane stabilizers, molecules that stimulate mitochondrial biogenesis, and others that target mitochondrial destruction by mitophagy. Some candidate therapies have been subjected to phase 2 or even phase 3 clinical trials, but none has been demonstrated unequivocally to have efficacy. Gene therapy involves introducing or modifying genetic material to correct or replace faulty genes. In mitochondrial diseases, this could involve targeting the nuclear or mtDNA to address mutations or defects. Challenges include the delivery of genetic material into the mitochondria (for mtDNA variants) and ensuring that the corrected genes are expressed properly (for both mtDNA and nuclear genes). Cellular and mitochondrial replacement therapeutic approaches involve replacing dysfunctional mitochondria within cells or replacing cells altogether, for example by hematopoietic stem cell or liver transplantation. Mitochondrial donation therapy using the techniques of maternal spindle cell or pronuclear transfer aims to prevent transmission of mtDNA disease by replacing mutated mtDNA with healthy mtDNA from a donor (174). This is currently the subject of a clinical trial in the UK, but results are yet to be reported.

Thus, this is an exciting time for mitochondrial medicine, with many novel therapies on the horizon. However, it is important to note that many of these approaches are still in the early stages of research, and their long-term efficacy and safety have yet to be fully established. Significant challenges in clinical trial design and execution remain to be overcome before effective treatments for this group of devastating conditions can finally be brought to the clinic (175).

## Conclusion

Endocrine dysfunction is observed in PMD with varying incidence depending on the specific mitochondrial disorder and the endocrine organ in question. Diabetes mellitus is the most frequently observed endocrine manifestation of mitochondrial disease, with the highest incidence observed in the MIDD phenotype associated with the m.3243A>G pathogenic mtDNA variant. Short stature, although not necessarily caused by growth hormone deficiency, and POI are also significant features of several PMDs. Male hypogonadism and hypogonadotropic hypogonadism are less certainly associated with PMD and require further cohort studies; however, reported numbers are so small that the challenge of establishing a causal relationship is likely to remain. Adrenal insufficiency and hypoparathyroidism are rare, but present in sufficient patients that they should be screened for in all patients with PMDs, especially certain at-risk groups such as patients with SLSMDs. Hypothyroidism is not commonly associated with PMD. The key to diagnosing a PMD in a patient presenting with endocrine disease is the high level of clinical suspicion that should be employed when dealing with atypical presentations, or when faced with apparently unrelated comorbidities. Mitochondrial disease is often synonymous with complex health needs, and prompt diagnosis facilitates the necessary screening and referral to multisystem health professionals that are likely to be needed.

## Abbreviations

CYP11A1: cholesterol side-chain cleavage enzyme
FGD: familial glucocorticoid deficiency
GDM: gestational diabetes mellitus
MELAS: mitochondrial encephalomyopathy, lactic acidosis, and stroke-like episodes
MERRF: myoclonic epilepsy with ragged red fibers
MIDD: maternally inherited diabetes and deafness
mtDNA: mitochondrial DNA
NAMDC: North American Mitochondrial Disease Consortium
NNT: nicotinamide nucleotide transhydrogenase
OXPHOS: oxidative phosphorylation
PEO: progressive external ophthalmoplegia
PMD: primary mitochondrial disorder
POI: premature ovarian insufficiency
ROS: reactive oxygen species
rRNA: ribosomal RNA
SLSMD: single large-scale mtDNA deletion
SNHL: sensorineural hearing loss
StAR: steroidogenic acute regulatory protein
TIM: translocase of the inner mitochondrial membrane
TOM: translocase of the outer mitochondrial membrane
tRNA: transfer RNA
TXNRD2: thioredoxin reductase 2
UPRmt: mitochondrial unfolded protein response
